# The Organic Turn: Coping With Pandemic and Non-pandemic Challenges by Integrating Evidence-, Theory-, Experience-, and Context-Based Knowledge in Advising Health Policy

**DOI:** 10.3389/fpubh.2021.727427

**Published:** 2021-11-24

**Authors:** Holger Pfaff, Jochen Schmitt

**Affiliations:** ^1^Faculty of Human Sciences, Faculty of Medicine and University Hospital Cologne, Institute of Medical Sociology, Health Services Research, and Rehabilitation Science, University of Cologne, Cologne, Germany; ^2^Center for Evidence-Based Healthcare, Medical Faculty Carl Gustav Carus, Technical University Dresden, Dresden, Germany

**Keywords:** evidence-based health policy, mechanistic vs. organic knowledge processing, experts, COVID-19, theory, agile science

## Abstract

The COVID-19 pandemic has posed an extraordinary challenge for public health and health policy. Questions have arisen concerning the main strategies to cope with this situation and the lessons to be learned from the pandemic. This conceptual paper aims to clarify these questions *via* sociological concepts. Regarding coping strategies used during the pandemic, there is a strong tendency for health policymakers to rely on expert knowledge rather than on evidence-based knowledge. This has caused the evidence-based healthcare community to respond to urgent demands for advice by rapidly processing new knowledge. Nonetheless, health policymakers still mainly rely on experts in making policy decisions. Our sociological analysis of this situation identified three lessons for coping with pandemic and non-pandemic health challenges: (1) the phenomenon of accelerating knowledge processing could be interpreted from the organizational innovation perspective as a shift from traditional mechanistic knowledge processing to more organic forms of knowledge processing. This can be described as an “organic turn.” (2) The return of experts is part of this organic turn and shows that experts provide both evidence-based knowledge as well as theoretical, experiential, and contextual knowledge. (3) Experts can use theory to expeditiously provide advice at times when there is limited evidence available and to provide complexity-reducing orientation for decisionmakers at times where knowledge production leads to an overload of knowledge; thus, evidence-based knowledge should be complemented by theory-based knowledge in a structured two-way interaction to obtain the most comprehensive and valid recommendations for health policy.

## Introduction

Modern health policy faces two problems. Firstly, in many areas of healthcare, the rapid accumulation of knowledge and evidence is barely manageable for policymakers. Secondly, technological or social innovations and new biological risks (e.g., COVID-19) have presented certain areas with new challenges, and the knowledge to cope with these situations is lacking. The COVID-19 pandemic is an example of a situation where both of these problems have arisen: the lack of knowledge at the outset (phase 1) was replaced after a few months by an exponential rise in knowledge production (phase 2). This rapid increase in evidence production exacerbated the problem of research waste (1) due to the unnecessary duplication of studies and poor study quality (2). In both phases, the knowledge deficit phase (phase 1) and the knowledge overload phase (phase 2), policymakers often need support with regard to the complex decisions they have to make.

In this paper, we argue that engaging experts and changing the process of knowledge engineering (the organic turn) are solutions to solve these problems. COVID-19 is an extreme example of the fast emergent natural or artificial phenomena health policymakers have to cope with. An analysis of the COVID-19 pandemic provides insights into the performance capacity of traditional knowledge-producing and knowledge-broking institutions, their clients (policy decisionmakers), and the efforts to cope with these new situations. We describe a change in the institutional coping pattern, which we propose to call the organic turn. The COVID-19 pandemic therefore represents a paradigm for change.

## Methods

We integrated three complementary methods to derive the lessons learned from the pandemic concerning the process of advising health policy in times of rapidly changing environments. The methods used include (a) a selective literature review on the lessons learned thus far, (b) an analysis of societal and scientific reactions to the pandemic, and (c) organizational and social theories that could explain the problem analyzed. The selective literature review revealed that there is a growing number of papers that discuss the lessons of the COVID-19 pandemic (3). These are mainly specific lessons (3–6), and there is a deficit in more general explanations and hence in more general strategic lessons. In our second step, we analyzed the predominant strategies for coping with the pandemic by scanning news about pandemic decision-making, especially in Germany. This analysis indicated that political decisionmakers had to make decisions without relying on evidence-based knowledge specific to COVID-19. In the beginning, politicians predominantly relied on scientific experts, particularly virologists, epidemiologists, and mathematical modeling experts (7–12). After having been called upon for help by politicians, the scientific community developed new, agile ways of assembling knowledge quickly (13–16). In our third step, we sought an established social and organizational theory that could explain the societal and scientific coping pattern identified. We scanned various social and organizational theories, especially the system theories of Talcott Parsons (17) and Niklas Luhmann (18), the bureaucratic theory of Max Weber (19), the resource dependency theory of Jeffrey Pfeffer (20, 21), and the contingency theory of Tom Burns and G.M. Stalker (22). We found that Burns and Stalker's contingency theory (22) delivered the best general explanation for what happened during the COVID-19 pandemic. Their distinction between mechanistic and organic systems best fits our outline of the societal and scientific patterns of coping with the pandemic by using expert advices, theoretical approaches and fast, less standardized knowledge processing.

## Results: Three Lessons Learned

This section presents three lessons learned from coping with the COVID-19 pandemic. Firstly, we are in the midst of the organic turn. Secondly, integrating evidence-, theory-, experience-, and context-based knowledge is part of this organic turn and often the task of experts. Thirdly, one of the main tasks of the future is to combine evidence-based and theory-based knowledge as part of the organic turn.

The first lesson is that the traditional form of standardized, bureaucratic knowledge production, review, and transfer (knowledge processing) is suitable for stable environments but not for unstable environments. After an analysis of the situation in relation to the mechanistic-organic concept of organizational sociology, we diagnosed the start of a transition away from mechanistic knowledge processing toward an organic approach. We call this the organic turn, and we define organic knowledge processing as an unbureaucratic, semi-formalized, semi-standardized and expert-based way of knowledge production, review and transfer. The second lesson is that the bureaucratic inner limits of evidence reviewing, and transferring in traditional institutions [e.g., National Institute for Health and Care Excellence (NICE) or Institute for Quality and Efficiency in Health Care (IQWiG)] was a main reason for the rise of experts during the pandemic. By bureaucratic limits we mean the formalized and standardized processes, rules and structures these institutions use to select high quality studies, to review knowledge and to prepare evidence-based decisions in health policy. The bureaucratic quality of these rules, structures and processes have their advantages, but they constrain the speed of knowledge processing in an extraordinary way. One characteristic of the organic turn is that experts are no longer at the bottom of the evidence grade system. Experts are an integral part of the agile coping structures of the healthcare system. The third lesson is that in times of change and instability, there is a need for theory and theory-based knowledge that complements evidence-based knowledge and provides urgent orientation when there are evidence deficits or knowledge overload.

### Lesson I: Coping With Rapid Change by Moving From Mechanistic to Organic Knowledge Processing—The Organic Turn

The predominance of mechanistic evidence production, review, and synthesis is not appropriate in unstable situations, such as during times of natural disruptions (e.g., COVID-19) and/or rapid technological developments (e.g., digital transformation). Attempts to cope with these new events (e.g., modeling, rapid reviews, living guidelines) can be interpreted from an organizational sociologist viewpoint as attempts to shift from mechanistic knowledge production, review, and transfer to an organic form of knowledge processing.

#### Pre-COVID-19: Predominance of Mechanistic Evidence Processing

Evidence-based health policy depends on institutions that screen and provide evidence for policy decisionmakers. These institutions, such as the German Institute for Quality and Efficiency in Healthcare (IQWiG) and the National Institute for Health Excellence (NICE) in the UK, provide general, evidence-based knowledge of preventive, therapeutic, and diagnostic measures (23). These institutions often receive assignments from political decision-making institutions (in Germany: Federal Joint Committee) to provide evidence-based knowledge to inform the decision-making process (23–25). Thus, most of these institutions play an advisory role for the government, as in France and Australia, or for government-like institutions like the Federal Joint Committee (G-BA) in Germany (23, 25). It is important to note that the knowledge and advice these institutions provide is based solely on sound empirical evidence and not on (a) the experiences of these institutions; (b) their theoretical knowledge; or (c) the social, economic, and political contexts of their countries. As such, evidence-based knowledge is mostly context-free. It is the responsibility of the recipients of evidence-based knowledge to add theoretical, experiential, and contextual knowledge into the decision-making process to arrive at a balanced and appropriate decision (26).

Traditional knowledge-reviewing and -synthesizing institutions such as the NICE (27, 28) or IQWiG (29–31) are characterized by formal processes for preparing reviews, syntheses, and critical appraisals, and by official and transparent documentation. These formal processes protect against criticism, legitimize these institutions' decisions, and make the decisions transparent (30, 31). Out of the functional perspective in sociology these formal procedures have the function to enhance the legitimization of these institutions and their decisions (“legitimation by procedure”) (32). When considering official state-governed institutions from the organizational sociology standpoint and particularly in relation to classic bureaucratic theory (33), these traditional evidence-synthesizing and evidence-reviewing institutions are deemed bureaucratic institutions. Weber defines bureaucratic institutions as a form of legal authority based on rules, norms, or procedures. They are characterized by formalized, hierarchical, and specialized bureaus of office, and they are standardized, rule-based, and impersonal (34). An alternative term for a bureaucratic organization is a mechanistic organization (35). This term stems from Burns and Stalker (22, 36). Mechanistic organizations are appropriate in stable environments and suitable for routine tasks. They use specialization, standardization, and formalization to increase effectiveness, transparency, and predictability, resulting in rigidly defined processes. The rigid and formal processes of NICE (15, 28) and IQWiG (29–31), for example, are reflective of the elements of mechanistic organizations. A mechanistic strategy increases safety, transparency, and quality, but it is time-consuming (15).

#### COVID-19 Forces Organic Forms of Knowledge Processing: The Organic Turn

During a pandemic, traditional evidence-reviewing institutions struggle with their bureaucratic inner limits (16), as do guideline-developing professional societies and disease control institutions (e.g., Center for Disease Control; Robert-Koch-Institute). They must cope with one of the main structural problems of knowledge processing, namely that it takes time to produce and process sound, evidence-based knowledge and to assure the quality of the required steps (15, 28). This traditional knowledge process chain requires time-consuming randomized controlled trials (RCTs) to study the effects of interventions, especially if they want to detect the middle- and long-term effects. It also takes time to synthesize and critically appraise the results of different studies and to agree—based on this knowledge—in a consensus decision on clinical guideline recommendations.

However, during a pandemic, time is crucial. Policy decisionmakers have to make far-reaching decisions quickly (37) and therefore require rapidly generated evidence-based knowledge (13). In time-sensitive situations, decisionmakers realize that traditional evidence-generating institutions are not able to create, sample, and review existing relevant knowledge in the short time (15).

The structures and processes of traditional evidence processing institutions are unable to adequately cope with rapidly changing situations such as the COVID-19 pandemic. It takes too long to obtain the desired results if the institutions follow their rules and procedures, leading to structural helplessness; to overcome this, these institutions attempt to accelerate knowledge generation, review, and transfer by implementing lean procedures. However, this endangers high quality standards (15).

The scientific community and traditional knowledge-processing institutions have developed a series of innovative ideas, concepts, and tools to shorten the time span needed for knowledge processing, including parallel instead of sequential testing (38), tools for accelerating the development of innovations (e.g., vaccines) (39), streamlining the review process in journals and the use of preprints and preprint journals (40), and tools such as rapid reviews (13, 37) and living reviews (41) to quicken evidence-reviewing processes. Further examples are the use of “rapid guidelines” (15) or “living guidelines” to quickly disseminate recommendations to physicians and hospitals (42, 43) and the use of simulation models as alternatives to time-consuming empirical studies (14). These are reliable steps to cope with time constraints while still assuring high-quality knowledge (see Table 1).

**Table 1:** Accelerating knowledge processing: tools and measures.

	**Knowledge processing phases**		
	**Pre knowledge production**	**Knowledge production**	**Post knowledge production**
Measures to speed up the knowledge-producing process	Speeding up - Ethical approval - Proposal reviewing - Research standardization (core outcome sets)	- Shortening the “impact time span” under study - Using modeling approaches	- Preprint - Rapid reviews - Living reviews - Living guidelines - Rapid communications

Speed-accelerating tools and inventions focus on activities around the core process of knowledge production. Accelerating ethical approvals and quickening the review process for project proposals are measures taken before knowledge production begins. For example, core outcome sets are being developed to standardize international study protocols as a prerequisite for pooling after conducting studies (44). Speeding up article reviews and evidence synthesis through rapid systematic reviews and knowledge dissemination using living guidelines are measures implemented after knowledge production (see Table 1).

The crucial problem with these rapid measures is quality assurance (2), and some scholars have questioned whether it could be guaranteed that rapid measures, such as preprints without reviews, rapid reviews, and living guidelines, produce the same quality as non-rapid measures (13, 15).

We hypothesize that rapid knowledge processing is a solution not only to the problems posed by the COVID-19 pandemic but also a paradigmatic solution for all rapidly emerging events and innovations, which are known as “fast moving research areas” (41). Fast-moving research areas rapidly develop new findings, new methods, new therapeutic or diagnostic tools, and new patents.

As mentioned, measures for accelerating knowledge processing could be framed using an established concept from organizational sociology: the mechanistic vs. organic systems dichotomy (22, 45). The central hypothesis of this concept is that mechanistic systems are suitable for stable environments and organic systems for unstable, changing environments (22, 46, 47).

We predict that we will see parallel structures of mechanistic and organic knowledge processing in the near future. Where an environment is stable, as is the case in less innovative parts of the health care system, mechanistic knowledge generation and distribution is useful (see Table 2). Mechanistic structures and processes are highly formalized and administratively authorized. Processes outside the authorized protocols are considered—out of the mechanistic perspective—as variances that must be reduced to increase predictability and effectiveness. Decision-making in mechanistic institutions is largely concerned with the use of predetermined criteria, rules, or procedures.

**Table 2:** The organic turn: moving from mechanistic to organic knowledge processing.

**Type of environment**	**Stable or slowly changing environment in healthcare**	**Rapidly changing environment in healthcare**
Type of knowledge processing	Mechanistic knowledge processing:	Organic knowledge processing:
	• Classic EbM or EbHP • Cochrane reviews • “Normal” guidelines • Formal rules and procedures • Standardization • Minor role of experts	• Simulation models • Rapid reviews • Rapid or living guidelines • Flexible rules and procedures • Semi-standardization • Importance of expert advice
Change in knowledge processing: the organic turn	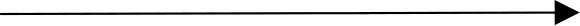	

*EbM: Evidence-based Medicine; EbHP: Evidence-based Health Policy*.

When an environment is unstable and changing, as it occurs with unpredictable events such as the COVID-19 pandemic or technological changes (e.g., therapeutic innovations), the opportunity increases for organic knowledge production, review, and transfer (see Table 2). It is important that organic systems value expertise (36). In organic institutions, formal and informal organizations overlap, commitment to the institution is strong, and professional values and beliefs substitute for formal hierarchy (22). In these systems, ambiguity is high, and the gains in flexibility come at the expense of structure (22, 36).

### Lesson II: Integrating Evidence-, Theory-, Experience-, and Context-Based Knowledge is Crucial in Advising Health Policy

The organic turn in knowledge production, review, and distribution is one of the reasons for the return of the expert during times of crisis because expertise is a crucial element of organic organizations. Experts have become central not only because expert advice is readily available and trusted during times of insecurity but also because expert knowledge serves hidden functions that were lost in the decades of evidence-based healthcare.

There are several definitions of the term “expert”. In this paper, an expert is defined as a person who possesses a specialist knowledge (48). An expert uses technical skills, and is—ideally—impartial (48). An important characteristic of experts is that they “mediate between the production of knowledge and its application; they define and interpret situations; and they set priorities for action” (48).

The original idea of the evidence-based movement was to retire the experts (49, 50) and to uncover their sin (51).

In recent years, and especially regarding COVID-19-related policy consulting, many observers have noticed a shift. Despite the anti-expert sentiment that exists in the general public (52), experts and their stockpiles of knowledge are increasingly in demand (53), and there is an impression that they are emerging from “retirement” (8, 54). Advocates of evidence-based medicine are therefore increasingly asking how the role of expert consultations for public policy should be interpreted and classified (55, 56). The issue is that at the onset of new, unexpected health-threatening events, there is typically no high-quality external evidence because the necessary data is lacking (55, 56). However, there is also an urgent need for action (57). Experts have therefore filled this evidence gap.

The need for experts remains even after implementing the accelerating tools because the problem of time-consuming evidence-based medicine persists, even if it is somewhat alleviated through rapid knowledge processing. Sound knowledge production still takes time. Even fast reviewing is too slow for politicians facing instability and rapid change. Following the formal procedures, the development of clinical guidelines still requires several months (43). The main obstacle of evidence-based science lies in the core of the knowledge generation process: the production of sound knowledge in scientific studies. It is extremely difficult to speed up studies. Time constraints are relevant during stable and unstable periods because in both cases researchers must wait to measure the effects of an intervention (“impact time”). Impact time is the time from the onset of an intervention until the desired outcome emerges. As an example of the problem posed by impact time, it takes more than 15 years to plan, conduct, and publish an RCT on colorectal carcinoma screening (58). Thus, in times of rapid change where there is no time to await the end of the impact time rapid advices of experts are necessary.

**Figure 1:**
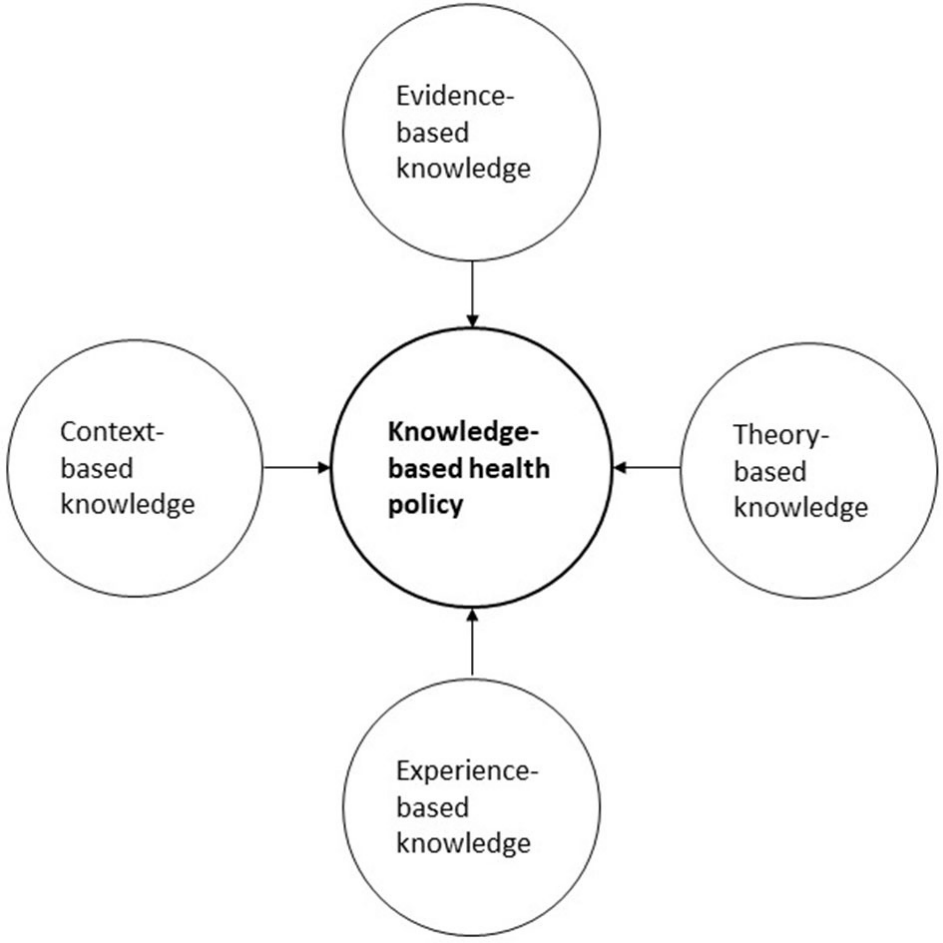
Knowledge-based health policy: knowledge components.

The organic turn in knowledge production, review, and transfer favors experts because experts fulfill additional functions beyond providing evidence-based knowledge. These additional functions include the provision of theoretical knowledge, experiential knowledge, and knowledge regarding the context of the planned interventions (see Table 3). The probability of high-quality decisions in health policy is highest if the decisions are transparently made based on the best knowledge available in all four knowledge domains: (1) evidence-, (2) theory-, (3) experience-, and (4) context-based knowledge. Using all four knowledge domains results in knowledge-based health policy (Figure 1). Knowledge-based health policy means to make health policy decisions based on (a) the best available external evidence from systematic research and sound mathematical modeling, (b) the best available theoretical knowledge, (c) the best experiential knowledge available, and (d) the best context-specific knowledge. Experts and policymakers should combine and synthesize these four knowledge components to arrive at balanced decisions in healthcare and health policy (see Table 3). The process of synthesizing and applying knowledge should be documented for transparency. Due to their ability to combine their tacit and experiential knowledge with other knowledge components, such as evidence-based knowledge and contextual knowledge, trusted experts play a central role in the process of knowledge transfer (59–64).

**Table 3:** Knowledge-based health policy: Functions of advising policy decisionmakers.

**No**.	**Functions of advising policy decisionmakers**	**The “ideal” expert or expert group**	**Evidence-reviewing and providing institutions**
1	Providing evidence-based knowledge	Yes	Yes
2	Providing theory-based knowledge	Yes	No
3	Providing experience-based knowledge	Yes	No
4	Providing context-based knowledge	Yes	Limited
5	Synthesizing and transferring the knowledge stemming from 1–4 to provide context-sensitive policy advice	Yes	No
6	Reflexive thinking	Yes	Limited
7	Timely provision of knowledge	Yes	Limited

To support decisionmakers in the decision-making process, experts and decision-supporting institutions should not only provide knowledge form these four knowledge domains (48, 65, 66). They should also provide knowledge synthesis, reflexive thinking and timely advice. Thus, we are able to identify seven categories of advices decision supporters can give to policymakers. Table 3 lists the seven functions that experts should engage in to maximize the quality and timeliness of policy decisions; this list is not exhaustive, but it contains the most important functions for creating sound and balanced knowledge-based health policy.

The first function of decision support (i.e., expert support) is to provide decisionmakers with the best available evidence-based knowledge by searching for, reviewing, and classifying clinical and public health research and then transferring this knowledge to decisionmakers (59–63). Knowledge is derived from observational studies and studies with quasi-experimental and experimental designs as well as from mathematical modeling (67–69).

The second function of decision support is to provide the best available theoretical knowledge pertaining to the decision to be made. Theoretical knowledge involves using established theories or models as a guide to health policy strategies. Lewin summarized the practical usefulness of theoretical knowledge with the quote “there is nothing so practical as a good theory” (70). Theoretical knowledge is one of the central domains of experts, and experts can provide theories that guide policymakers (71, 72).

The third function of experts is the provision of experience-based knowledge relevant to the decision. Experiential knowledge denotes the proficiency and judgement that experts, advising institutions, or decisionmakers acquire through experience and practice. Expert knowledge comprises—among other forms of knowledge—experience-based tacit knowledge and experience-based intuition (61, 73–75).

The fourth function of expert advice is to provide context-based knowledge to tailor evidence-based knowledge to the situation. Context-based knowledge pertains to the physical-chemical, biological, and social situations and possible changes in those situations (18, 76, 77). One important aspect of the context is knowledge of the processes, legal frames, and rules that have to be considered in decision-making (72).

The fifth function of decision support is to synthesize evidence-based, theoretical, experiential, and contextual knowledge and to adapt it to the decisionmakers (48). Experts can provide scientific information, convince the decisionmaker to consider a single best option, or adapt scientific information to decisionmakers preferences to reduce the choices provided (78).

The sixth function of decision support is to stimulate reflexive thinking (79). Reflexive thinking involves evaluating the direct and indirect costs and the “unanticipated consequences” (80) of the decisions made. In addition, reflexive thinking means learning about contextual nuances and possible interactions between the intervention and the context (77, 81). From the organizational learning perspective (82, 83), reflexive thinking involves single-, double-, triple-, and quadruple-loop learning (82, 84–86) as well as unlearning processes (87). All these learning types can be found on the individual, collective, and organizational level. Single-loop learning is trial-and-error learning without questioning the policies, basic assumptions, and goals underlying the trial-and-error actions, while double-loop learning involves questioning these factors (82). Triple-loop learning means learning about single- and double-loop learning (88). Triple-loop learning also involves building a learning infrastructure that connects local clusters of learning (89). Quadruple-loop learning involves continuous, context-specific learning to cope with uncertainty and complexity by revising, redefining, and expanding triple-loop learning (90). Articles on the lessons learned from the COVID-19 pandemic are examples of reflexive thinking. In a recent article comparing US and South Korean pandemic strategies, researchers demonstrated that single- and double-loop learning were essential to cope successfully with the COVID-19 pandemic (91).

The seventh function of decision support is to provide the required knowledge in a timely and easy-to-understand fashion (92, 93). Individual experts or expert groups represent the most agile knowledge sources. They are flexible and accessible and they are able to combine all four knowledge components.

As Table 3 shows, the established evidence-based medicine (EbM) institutions cover the most important function of providing empirical evidence. Some institutions derive recommendations for clinical practice based on this evidence, often using the Grading of Recommendations Assessment, Development and Evaluation process (GRADE) as a framework (94). However, these institutions completely fulfill only one of the functions required to guarantee high-quality decisions. The other functions are not or only incompletely fulfilled. In a world of rapid medical change, this gap is increasingly being filled by experts. In sum, expert knowledge has four dimensions: evidence-, theory-, experience-, and context-based knowledge (Figure 1). Ideally, experts or expert groups have sufficient theoretical knowledge, are up to date with current evidence regarding the given problem, and have enough experience to use their context-specific knowledge to integrate all four aspects of knowledge and to apply them to the situation at hand. In short, the advantage of evidence-based experts and expert groups compared to evidence-based government institutions is the use of four-dimensional knowledge that can be applied to a specific situation in a timely and reflexive manner.

### Lesson III: Integrating Theory-Based Knowledge and Evidence-Based Knowledge as a Special Challenge in the Organic Turn—The Scientific Knowledge Triangle

The third lesson indicates that integrating theory-based knowledge and evidence-based knowledge is a necessary component of the organic turn. Because this integration poses a special challenge, it is necessary to examine this task more closely.

Scholars who study the use of theory in health services research and especially in implementation research have stated that there is an underutilization of theory in health services research in general and in implementation science in particular (95); this is already a problem in normal times, but in rapidly changing times, this underutilization could contribute to disorientation in health policy with regard to the right starting-points, the right direction and the right plans.

When there is no evidence, as is the case at the onset of new situations, established theories can fill the knowledge gap and be used to advise health policy by indicating the starting-points for plans and measures. When there is an exponential growth in evidence, a lack of theories can hinder (a) the meaningful integration of existing evidence-based knowledge elements into a broader picture, (b) the explanation and prediction of complex phenomena, and (c) the guidance of evidence-based research.

Hence, the question arises as to why theories play no official role in evidence-based health policy and evidence-based healthcare; one possible reason for this is that there is a lack of quality grading for theories comparable to the quality grading of empirical studies in evidence-based healthcare. Therefore, we propose grading the quality of theories on a meta-level. With meta-level we mean assessing theories not by the quality of the content, but rather by formal criteria, like the spread and acceptance of theory in science.

There are different gradings for empirical evidence (96). However, to our knowledge, no clear-cut, standard, quality-oriented ranking of theories exists that differentiates between “low quality” and “high quality” theories similar to the hierarchical levels in evidence-based medicine. The quality of theories is an important topic in the areas of artificial intelligence, machine learning, and big data (97–99), but it is less discussed in health services research, though there are a few exceptions to this, such as in the realm of theories of health behavior changes (100, 101).

In general, there is no consensus in the literature on how to define and assess the quality of a theory. The measure of quality depends on the paradigm used, such as positivist, post-positivist, critical theory, or constructivist paradigm (97, 102, 103). To overcome this problem, we investigated the quality of theories from two formal perspectives. The first perspective is the post-positivist perspective, which has the advantage of being compatible with the positivist approach of evidence-based medicine but also allows the inclusion of qualitative results and probabilistic hypotheses (102–105). The second perspective is the constructivist perspective, which has the advantage of considering the process of the social construction of knowledge in a scientific community (106–109). Based on these two perspectives, we propose the use of two criteria to grade the quality of a theory: (1) acceptance of the theory in the scientific community and (2) empirical confirmation of the theory or parts of the theory. From a constructivist point of view, the acceptance of a theory in the scientific community, which can be measured approximately for example by how often the theory is cited in the literature (98) or by how often the theory is used in the scientific community (95, 109), is an indicator of the intersubjective quality and usefulness of the theory. From the post-positivist point of view, the quality of a theory depends on the successful testing of the theory or parts of the theory (e.g., single hypothesis) in empirical, evidence-based studies.

**Figure 2:**
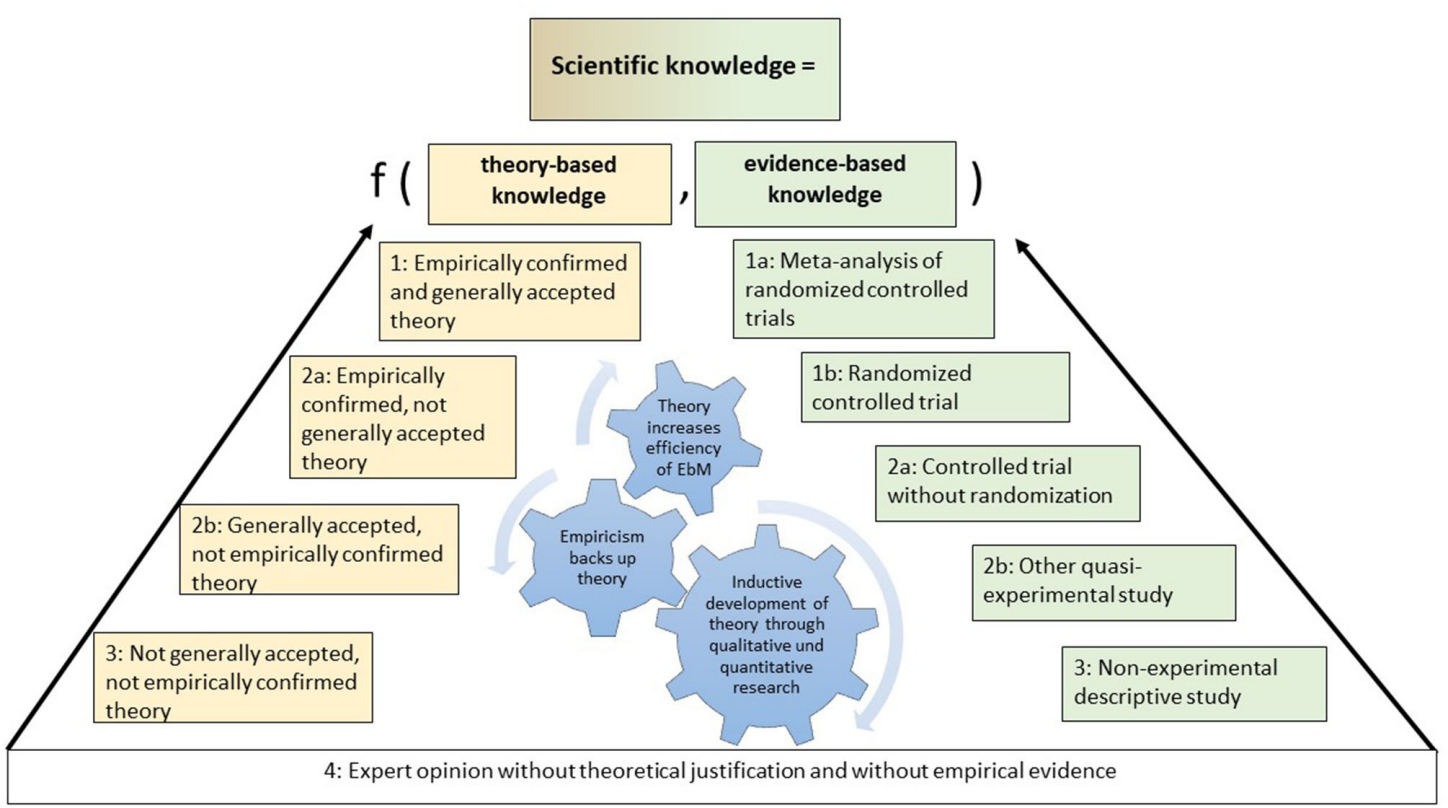
Scientific knowledge triangle: integrating evidence-based and theory-based knowledge.

To apply these two criteria to theories in the health sciences, we propose the grading of theories as shown in the “scientific knowledge triangle” in Figure 2. The formal, meta-analytical hierarchy of theories on the left side of the triangle complements the evidence-based knowledge hierarchy that comprises the right side of the triangle, thereby creating the scientific knowledge triangle.

We used the grades of evidence listed by Shekelle et al. (110) to grade evidence-based knowledge. In the following section, we focus on the left side of this scientific knowledge triangle.

The base of the scientific knowledge triangle is the opinions of experts, which are not evidence-based and do not have a theoretical basis or justification. Expert opinions alone are classified as having little or no recommendation strength. Two sides emanate from this “zero base”: the evidence-based side on the right and the theory-based side on the left.

Based on the identified criteria for ranking the formal quality of a theory, the first level of theoretical knowledge is achieved if there is a systematic, internally consistent theory, model, framework, or concept that has not yet been accepted by a relevant part of the scientific community and has not been empirically confirmed (level 3).

The next formal quality level of a theory, level 2b, is reached if the theoretical knowledge is based on a systematic, internally consistent theory, model, framework, or concept that has been accepted by a relevant part of the scientific community but has not been empirically confirmed; an example of this is the sociological systems theory (18, 111).

Because of the preference for empirical evidence over consensus, the next formal quality level of theoretical knowledge (level 2a) is attained when there is a systematic, internally consistent theory, model, framework, or concept that has not been accepted by a relevant part of the scientific community but has been empirically confirmed (level 2a in Figure 2). An example of this is the use of social learning theory (112, 113) within implementation science, where this theory is not really broadly accepted or used (95).

The highest level of theoretical knowledge (level 1) is attained when a systematic, internally consistent theory, model, framework, or concept has been accepted by a relevant part of the scientific community and has been empirically confirmed (level 1 in Figure 2); examples of this are the theory of planned behavior in the science of behavior change (100, 114–116) and in management science (98).

The evidence-based approach would benefit from the addition of a theory-driven approach. In the various fields of medicine, public health, psychology, and the social sciences, the overarching goal should be to implement both forms of knowledge generation to better explain and understand reality. Combining evidence-based and theory-based knowledge creates a body of scientific understanding that can inform health policy in a sound and balanced way.

The relationship between the two types of knowledge is reciprocal. Theory can inform evidence-based knowledge and vice versa. For example, one of the most important functions of theories is to guide the direction and process of empirical research (orientation function), such as by planning experiments in a theory-based manner from the outset (117) or by using logic models (118). Another path of cross-fertilization runs from evidence-based knowledge to theory-based knowledge, which is also known as the inductive approach. A classic form of empirical theory-building is given in some forms of qualitative research (119). The third path of cross-fertilization is the classic approach to build a theory by testing parts of the theory with empirical research. If a trial confirms a hypothesis, the theory-based knowledge is further supported by empirical work with reliable evidence. However, if the RCT does not confirm an important hypothesis of the theory, this represents a classic example of the falsification of a hypothesis (120) that puts the quality of the theory used into question.

## Discussion

This paper aimed to demonstrate that the COVID-19 pandemic can act as a starting point for an organic turn in evidence-based science. This proposition is derived from the first lesson discussed, namely that accelerating the speed of knowledge production, review, and transfer leads to a paradigm shift wherein the traditional mechanistic approach to knowledge processing is exchanged for a more organic approach. Some scholars may argue that accelerating speed does not indicate a fundamental shift away from traditional procedures (41). We maintain that accelerating speed is only one element in the broader reaction of science and health policy to highly changeable environmental phenomena. Another important element is the rise of experts. If this shift is sustainable, the overall picture of moving from bureaucratic procedures to more agile forms of knowledge processing represents a turn.

The second lesson is that the rise of multi-function experts during the pandemic demonstrates that organic knowledge processing requires the integration of evidence-, theory-, experience-, and context-based knowledge to advise health policy. One argument against this stance could be that experience-based knowledge must be excluded from the list of important knowledge components because it is subjective and not objectively verifiable. We believe that experts' experiences (e.g., experience of former pandemics) are, next to theory, one of the most agile forms of knowledge components and one where learning takes place. Experience is the result of individual and collective learning, therefore it is a useful, not neglectable form of knowledge. These agile components of learning are necessary in science to adapt and react quickly to new events and conditions. To exclude experience would mean to exclude intersubjective, subjective and tacit learning from scientific advice (75, 121). Another counterargument against our conclusions is that providing context-based knowledge is not the task of science but of health policy or healthcare decisionmakers. However, according to complexity science and implementation science, context should be included into scientific advice, because most of the evidence-based interventions are context-dependent (77). Context is a possible moderator or mediator of the intervention-outcome relationship and has therefore to be integrated into research, research designs, and policy advice (77, 122).

The third lesson is that integrating theory-based knowledge and evidence-based knowledge as part of the organic turn poses a special challenge. We therefore proposed the scientific knowledge triangle. Some scholars may argue that theory is already part of evidence-based healthcare and evidence-based medicine, as in the case of using logic models to legitimize interventions evaluated by RCT designs. Indeed, using logic models exemplifies integrating evidence-based and theory-based perspectives. Additionally, the hierarchy of theories proposed can contribute to improved logic models by identifying more high-quality theories instead of simple “if-then” constructions when planning RCTs. Another counterargument against our conclusions could be that it is extremely difficult to distinguish between the different quality levels of the theories. To facilitate distinguishing between the different quality levels of theories, there is a need to operationalize in the future what is meant by “empirically proven” or by “acceptance within the scientific community,” specifically.

The proposed three lessons provide a solid basis to make comprehensive and valid recommendations for health policy in unstable situations such as pandemics and digital transformation.

## Data Availability Statement

The original contributions presented in the study are included in the article, further inquiries can be directed to the corresponding author.

## Author Contributions

HP and JS conceptualized, wrote, and reviewed the manuscript. Both authors contributed to the article and approved the submitted version.

## Conflict of Interest

The authors declare that the research was conducted in the absence of any commercial or financial relationships that could be construed as a potential conflict of interest.

## Publisher's Note

All claims expressed in this article are solely those of the authors and do not necessarily represent those of their affiliated organizations, or those of the publisher, the editors and the reviewers. Any product that may be evaluated in this article, or claim that may be made by its manufacturer, is not guaranteed or endorsed by the publisher.
